# Affective polarization of space under imminent physical threat: Self-reported affect, physiology, and social responding across two experiments

**DOI:** 10.1371/journal.pone.0356294

**Published:** 2026-09-21

**Authors:** Till Kastendieck, Ursula Hess, Christophe Blaison

**Affiliations:** 1 Department of Psychology, Social and Organizational Psychology, Humboldt-Universität zu Berlin, Berlin, Germany; 2 Laboratoire de Psychologie Sociale, Université Paris Cité, Paris, France; New York University Abu Dhabi, UNITED ARAB EMIRATES

## Abstract

When humans navigate through their social and spatial surroundings, they construct affective representations of the spatial context that shape this navigation. The theory of affective judgment in spatial context specifies how people build these representations. We present two laboratory experiments (N = 121 and N = 254) that lend more ecological validity to this line of inquiry. In a modified threat-of-shock paradigm, participants experienced an actual physical threat at one location of an environment depicted on a computer screen. Like in previous online experiments, we observed that, compared to a control condition, participants reported that the threat at this location (termed hotspot, an affectively salient location in space) colored the surroundings negatively with high subjectively perceived arousal at close distance whereas it colored the surroundings positively with low subjectively perceived arousal farther away (i.e., an affective polarization of space). However, this polarization was not mirrored by physiological indices of affect, even though electrodermal and cardiac activity lent support to the idea that participants actually were more threatened closer to the hotspot. Furthermore, we could not find evidence for affective polarization elicited by the threat affecting the affective empathy in response to strangers shown at the different locations. Thus, whereas the subjective experience of affective polarization of space seems robust across different operationalizations, we did not find evidence that this subjective experience is reflected in underlying physiology or that affective empathy is moderated by distance to a threatening location.

People do not navigate through a purely geometric world. In addition to physical layout, they also evaluate places in terms of affective qualities such as attractiveness, threat, or comfort. This affective dimension is visible in a wide range of place-related judgments: people attribute stereotypical characteristics to spaces associated with specific social groups [1], spaces can affect the people who live there [2,3], and places shape prejudice, discrimination, identity, and expectations about who belongs where [4–7]. Because such evaluations orient approach and avoidance, they tie the affective dimension of spaces to decisions about movements and social engagement.

More broadly, affect provides information that is central in guiding evaluation, decision, and behavior [8–10]. Work on affective judgment in spatial context examines how people experience environments affectively as a function of the people and objects they contain, that is, how affect becomes attached to locations and not only to discrete events ([11–16]; for a review, see [16]). In previous work, this idea was formalized in spatial terms as an affective field, that is, a representation that maps anticipated affect onto physical space and guides approach toward places associated with positive affect and avoidance of places associated with negative affect [16]. One robust finding from this line of research is that affectively salient elements in the spatial context, or “hotspots,” cause “affective polarization of space” [13]. Theoretical accounts of this pattern appeal to comparison dynamics in judgment. The mechanism underlying this polarization can be understood through the inclusion-exclusion model of judgment [17]: the evaluation of a target location depends on whether contextual information is included in or excluded from the target’s mental representation. Spatial proximity favors inclusion, so nearby locations assimilate toward the hotspot’s affective tone; spatial distance favors exclusion, so distant locations serve as comparison standards and contrast away from it. Consistent with this account, hotspots elicit an assimilation effect nearby but a contrast effect farther away. For instance, a stigmatized neighborhood taints its immediate surroundings (an assimilation effect) but makes the surroundings farther away look more attractive compared to a control condition without a hotspot (a contrast effect; [14]. Conversely, a nice park makes the surroundings nearby appear more attractive (assimilation) but makes the surroundings farther away look less attractive compared to a control condition (contrast; [14].

Blaison, Fayant, and Hess [11] gauged the practical magnitude of spatial contrast by asking U.S. participants to report how much monthly rent they would be willing to pay for an apartment at the location farthest from an unsafe housing block, relative to conditions with a nearby park or no hotspot. The contrast effect on rent at that distance (about $69 higher than control) was statistically comparable to the assimilation effect of placing a pleasant park near the judged location (about $72), corresponding to roughly 10–13% higher rents in both cases. This result shows that spatial contrast is not a negligible side effect but can be economically meaningful in the same range as a clearly attractive amenity.

Most evidence for assimilation and contrast comes, however, from verbal vignettes and schematic neighborhoods (e.g., [11–13]). Such designs allow strong tests of the inclusion–exclusion logic but capture anticipated affect under hypothetical conditions rather than first-person exposure to an ongoing threat. Accordingly, whether the subjective gradient replicates under immersive presentation with localized aversive stimulation remains an empirical question, as does whether physiology, like autonomic activation, and facial-motor responses track that gradient or reflect processes that are only partially aligned with self-reported valence and arousal. The experiments reported here extend this line of research in two steps. Experiment 1 tested whether affective polarization of space generalizes to a laboratory paradigm involving actual physical threat and whether the effect that has so far been studied in the domain of subjective experience measures extends to physiological responses as measured by electromyography, electrocardiography, and electrodermal activity. Experiment 2 then examined whether the affective experience elicited by a hotspot also influences responses to strangers encountered in physical space.

## Experiments

### Experiment 1

#### Introduction

##### Affective polarization of space and subjective and physiological indicators of affect.

The core observation of the affective polarization of space is a change in affect as a function of distance from a hotspot. In previous research that assessed this effect via self-report using a vignette approach, participants were informed about the presence of a hotspot via a verbal description and a survey view of a neighborhood (e.g., [13]). This is a limitation because it remains possible that affective polarization of space occurs only when participants engage in a distanced mental projection of anticipated affect. Thus, to increase ecological validity, the present research puts participants in a first-person perspective and uses an actual threat to induce negative affect at the hotspot location. A second extension over previous studies in this line of research is that we assessed facial responses via facial electromyography (EMG). Surface facial EMG over the zygomaticus major, the muscle that lifts the corners of the mouth often associated with smiling, and corrugator supercilii, the brow-furrowing muscle often associated with frowning, are often associated with positive versus negative affect [18] and, when recorded under standard conditions, shows adequate reliability [19]. This method is often associated with being able to differentiate the valence and intensity of affective reactions toward visual emotional stimuli (e.g., [20]; for reviews, see [21,22]), even when these stimuli are presented subliminally [23,24], or when no change in facial display is observed with the naked eye [25]. However, EMG activation can have multiple reasons (e.g., corrugator activity due to squinting) and is therefore no direct proxy of valence; rather, within the scope of what is plausible in a certain experimental context, it is useful to consider EMG patterns, such as low corrugator activity paired with high zygomaticus and orbicularis oculi activity (and its inverse) as context-constrained physiological correlates of positive and negative affect (see also, [19]). Moreover, autonomic activation was assessed using electrodermal and cardiac measures. Importantly, these measures reflect nervous system responses rather than subjective arousal directly: skin conductance is a relatively pure index of sympathetic activation that is reliably elevated by threat [26–29], whereas heart rate is jointly controlled by sympathetic and parasympathetic activity [30]. For Experiment 1, we hypothesized an assimilation effect such that compared to the control condition, participants would report significantly more negative valence and higher arousal at the closest location from the hotspot. We hypothesized also a contrast effect such that compared to the control condition, participants would report significantly more positive valence and less arousal at the farthest location from the hotspot. Moreover, we explored whether the physiological measures would mirror the pattern of the subjective ratings. That is, compared to the control condition, we expected significantly more facial activity typically associated with negative affect (i.e., high activity in the corrugator supercilii; low activity in the zygomaticus major; high activity in the lateral orbicularis oculi, the muscle around the outer eye that produces the eye-crinkling often seen in smiles) as well as significantly higher skin conductance and higher heart rate at the closest location from the hotspot, combined with significantly more facial activity reflecting positive affect (i.e., low corrugator activity; high zygomaticus and orbicularis oculi lateralis activity) as well as significantly lower skin conductance and lower heart rate at the farthest location from the hotspot. We emphasize the exploratory character of the assumptions for the physiological measures as the activity of these measures can result from a variety of reasons, such as attentional effort, visual sampling, concentration, emotional and social display, or task demands.

#### Method

##### Overview.

In the experimental group, we induced a negative hotspot experimentally via a modified threat-of-shock paradigm [31]. Participants were immersed in a 3D neighborhood with a first-person perspective depicted on a large computer screen. The screen first showed a grim-looking housing block. At this location, participants saw superimposed videos (tabloid-media videos) of violent gang activity paired with a number of electrical stimulations at participants’ individually calibrated shock intensity framed so as to represent the “immediate threat of a physical assault” (this stimulus compound was the negative hotspot). Next, the participants were told that the likelihood of receiving an electrical stimulation (i.e., getting “assaulted”) decreased with increasing distance to the hotspot. Each participant was then visually relocated to three locations at increasing distance from the hotspot. This procedure is akin to real life where peoples’ belief that something bad may happen decreases with distance from a threatening location. The procedure in the control condition was identical except that there was neither a hotspot (and no electric stimulation whatsoever), that is, participants saw a neutral location at three increasingly remote distances from a first similarly looking location.

##### Participants.

A total of 121 (81 women and 38 men, 2 gender unknown) healthy German volunteers in the age range between 18 and 45 years (*M* = 22.03, *SD* = 1.27) participated in Experiment 1. Of these, 13.2% were psychology students, 42.1% were students from other fields and 43% were non-students, 1.7% did not report this information. All participants provided informed consent and received monetary compensation (12€/h) or course credit (psychology students). Depending on the measure, a small number of participants had to be excluded because of clerical error, technical failure, task disengagement, or excessive artifacts (see respective R script reports for analysis-specific sample sizes). Ethical approval was obtained from the institutional review board (IRB) of the Humboldt-Universität zu Berlin (proposal number #2015−30). Written informed consent was obtained for all participants. The data collection went from 1/17/2017–7/24/2017.

##### Design and procedure.

**Fig 1:**
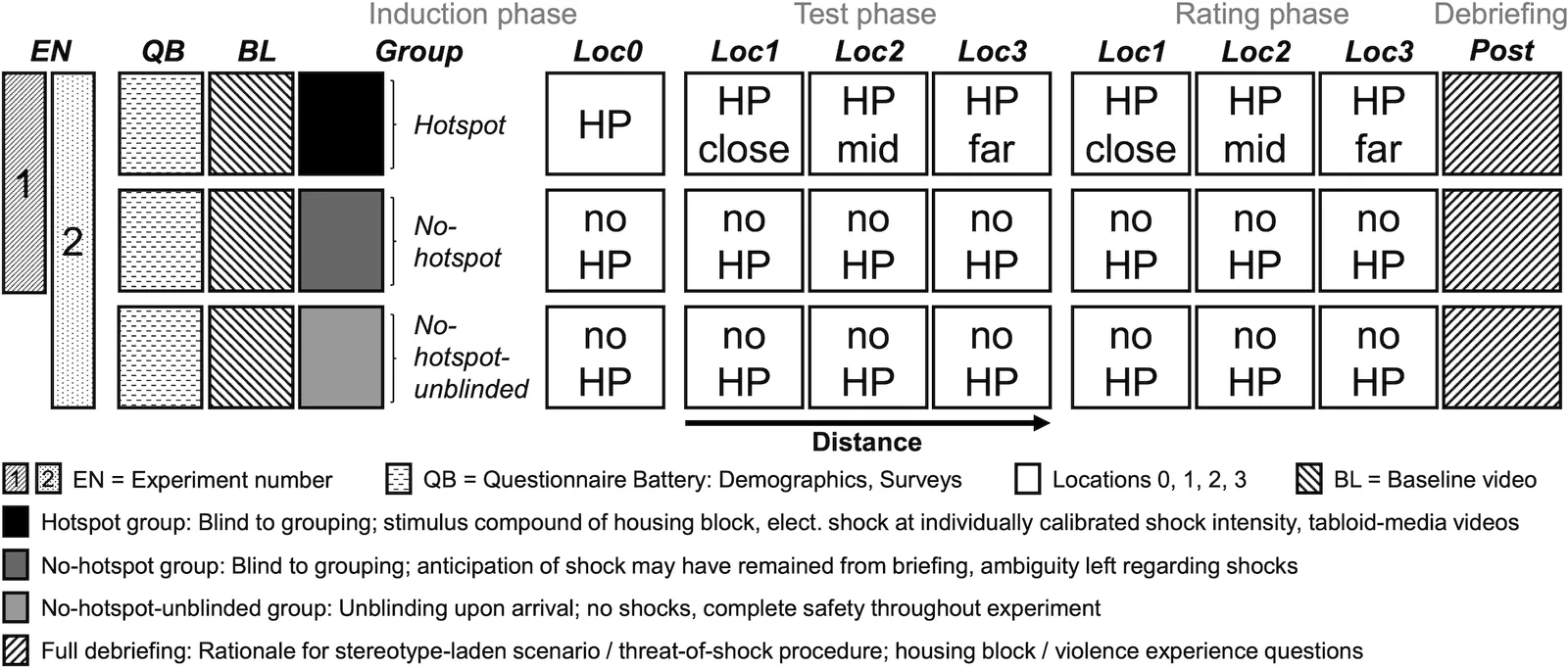
Overview of experimental setup.

Please see Fig 1 for a depiction of the procedure of Experiment 1.

###### Procedure of experiment 1.

**Fig 2:**
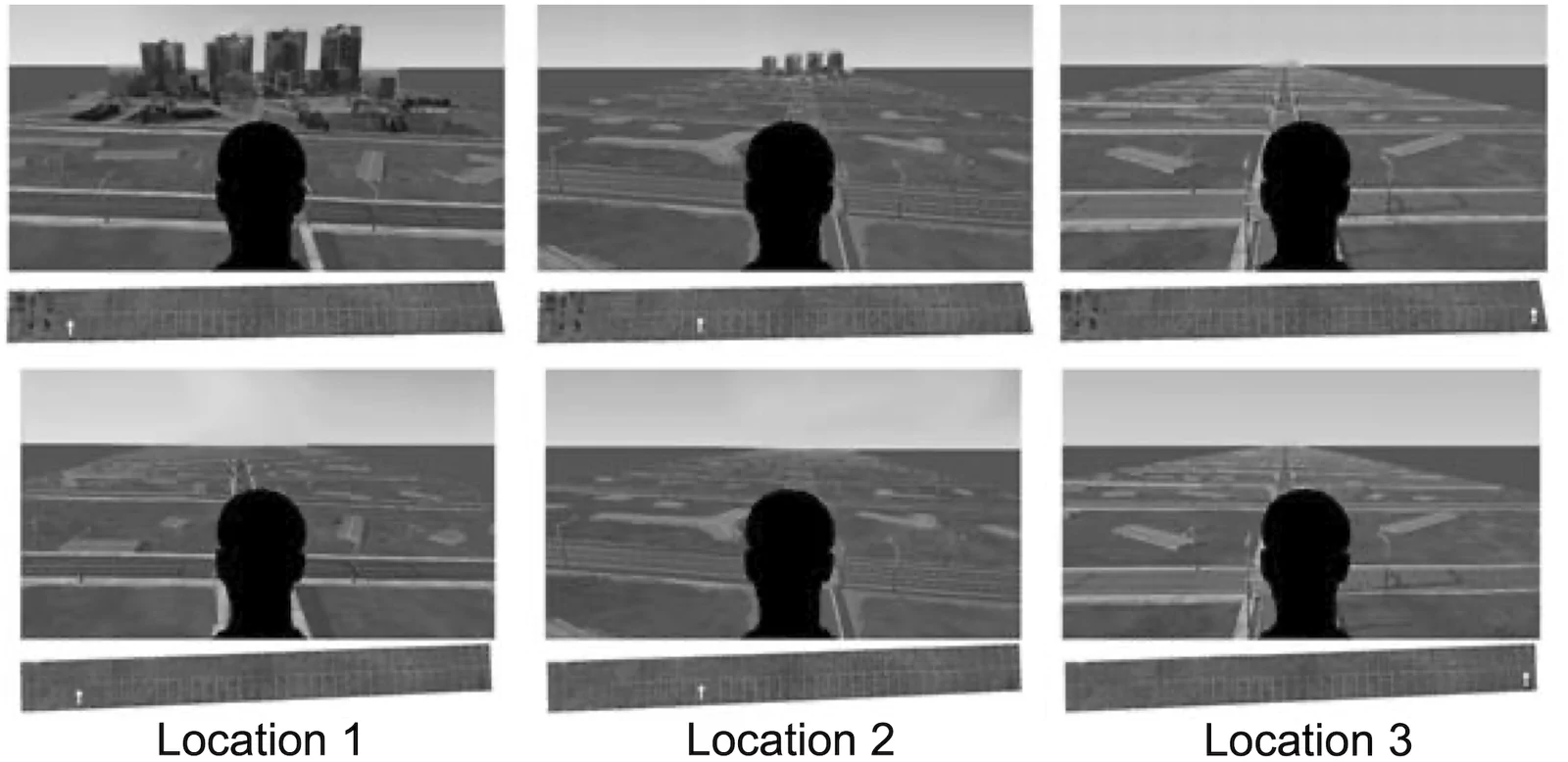
Overview of visual background material. Note: The figure shows the different locations for the different conditions (from close to far), which were presented to the participants in the experiment. Locations in the top row were presented to the experimental group (with visual cue of hotspot), whereas the locations in the bottom row were presented in the two control conditions (without visual cue of hotspot). Facial stimuli shown in experiment 2 with permission from the FACES database [32] rights owners were superimposed onto the scenes like the ghost actors but are not depicted. We used an elevated perspective because it was the most feasible and informative way to present the experimental scenes. The stimuli were generated using the build-modus in the SimCity PC-game, which was the most convenient and suitable program available to us at the time for creating a grim-looking neighborhood, but it did not allow a more flexible street-level perspective. At the same time, the elevated view had an important advantage: it made the spatial arrangement of the scene visible and enabled participants to estimate the distance between their position and the housing project (or the alternative location in the control group) more easily.

For ethical purposes, all the participants were informed that they might receive electrical stimulations depending on the experimental condition they would be randomly assigned to. The data collection took place in an artificially lighted, sound-attenuated, and electrically shielded room (Mean temperature 22.03, *SD* = 1.27; mean humidity 33.47, *SD* = 7.47). Participants sat in front of a computer screen in a comfortable armchair and first completed paper-and-pencil documents (informed consent, questionnaire). Then electrodes were attached and participants watched a relaxing three-minute baseline video (Baker Beach, San Francisco). After the baseline recording, the experimenter re-entered the room and gave further instructions. Participants were randomly assigned to one of the two groups. The study had a mixed design with presence of the hotspot as a between-subjects factor (two levels: hotspot vs no hotspot) and distance (location 0, 1, 2, 3) as a within-subjects factor (see Fig 1). In the hotspot group, participants were asked to determine their individually calibrated shock intensity for an unpleasant but not yet painful electrical stimulation (a common framing in a threat-of-shock paradigm). This individually calibrated shock intensity reflected the electrical stimulation value that participants reached following the instruction that the experimenter should put the electrical stimulation device to a level that aligned with the unpleasant-not-painful instruction. The electrical stimulations were applied with a Grass SD 9 Stimulator (Grass Technologies, Inc.) The duration of the shock was 12 ms with a delay of 10 ms, the voltage was increased in 5 V steps until the individual tolerance threshold was reached. Mean voltage in the sample was 58.3 V (*SD* =26.14, Min = 15, Max = 100). After that, the experimenter left the room and the computer task continued with physiological data being recorded throughout the rest of the experiment. The participants in the hotspot group proceeded with the hotspot procedure (induction phase) intended to induce negatively valenced feelings with high arousal toward the negative hotspot. This phase was composed of six trials that each lasted thirty seconds. Four of the six trials included shocks that were randomly triggered. During the trials, the participants saw a threatening video that was superimposed on the depiction of a housing block composed of dilapidated residential towers (i.e., location 0). The videos showed alleged gang members or street fighting scenes. In the subsequent test phase, participants were informed that they would be moved to locations that were increasingly remote from the negative hotspot, and that the likelihood of getting shocked (i.e., “assaulted”) would decrease “like in real life, when the likelihood of being assaulted decreases with increasing distance to a dangerous area” (wording in instruction). A fixation cross appeared for 1 s before participants were presented with the increasingly remote locations (i.e., locations 1, 2, and 3), which appeared for 30 s (see Fig 2). To avoid confounds due to the effect of particular other buildings, we told participants that the neighborhood was under construction. So, the only buildings in the area were the towers of the negative hotspot. As the towers of the negative hotspot could be seen from a distance, they served as reference points for the estimation of distance, a main reason why this scenario was used. Nevertheless, to help the participants to locate themselves mentally in the spatial context, we presented a satellite view from above along the immersed view (see Fig 2). At each position, an arrow on the satellite view indicated the position of the participant.

The procedure for the no-hotspot group was the same except that the participants were not exposed to the negative affect induction. Instead, they were just presented with a neighborhood under construction without any visually salient location (see Fig 2, lower panel). Furthermore, as an equivalent to the shock-tolerance-threshold procedure, they performed with the experimenter a bogus Weber-Fechnerian weight-discrimination task after the baseline video. In this task, the participants were asked to detect the threshold at which they could perceive a difference in weight. The task was conducted for a similar amount of time as the shock procedure in the hotspot group (3–5 minutes). Everything else was identical to the hotspot condition. The participants in the no-hotspot group were kept blind to the condition that they were randomly assigned to until the end of the experiment. At the end of the experiment, participants were debriefed. During debriefing, the experimenter explained the rationale for using residential towers as negative hotspots and worked toward deconstructing the negative stereotype toward this type of habitat. Participants were then thanked and compensated.

##### Measures.

###### Self-reported valence and arousal in response to locations.

Subjective valence and arousal elicited by the negative hotspot and the three remote locations were assessed via a 9-point scale adaptation of the self-assessment manikin (SAM; [33]. The same valence and arousal scales as for places were also used for the feelings elicited by the electrical stimulation in the experimental group.

###### Electrodermal activity.

For electrodermal activity, 8 mm Ag/AgCl electrodes filled with a neutral paste attached to a MindWare BioNex 8-channel amplifier were placed at the central phalanges of the index finger and middle finger of the left hand. As an indicator of activation in the sympathetic branch of the autonomic nervous system, the mean skin conductance level (SCL) in microsiemens (µS) across the 180 s of the induction phase, and across the 30 s of each remote location was corrected with the 3 m mean baseline SCL (cf. the recommended range of 2–4 m by Braithwaite et al., [34]. The detection of minimum threshold skin conductance reaction (SCR) was set to  .01 µS. The latency window for detection of SCRs was set to 1–3 s. Artifacts and superimposed skin conductance reactions were cleaned manually with the EDA module of MindWare BioLab.

###### Facial electromyography.

For facial electromyography (EMG), participants’ skin was cleaned using alcohol pads, scrub, and a cosmetic brush. We used 4 mm Ag/AgCl electrodes and the impedance was kept below 25 kΩ wherever possible. Muscle sites used were corrugator supercilii (typically associated with frowning, brow furrow), frontalis pars medialis (raising inner eyebrows), zygomaticus major (pulling lip corners as in a smile), orbicularis oculi lateralis [raising cheeks, often resulting in wrinkles around eyes, associated with (Duchenne) smile], auricularis posterior and orbicularis oculi medialis (the latter two intended for startle reflex, discarded from further investigation due to a programming error). Facial EMG data was recorded with a sampling rate of 1000 Hz with a 30–300 Hz band filter and a 50 Hz notch filter. The signal was offline rectified and smoothed with a 5 Hz lowpass filter. The raw EMG scores for each participant were binned to 1 s and were corrected with the mean of the last 90 s of the baseline period to account for interindividual differences in baseline muscle tension and univariate outliers (via adjusted outlyingness, [35] were removed. Individual muscle level scores were square root transformed to attenuate the skewness commonly found in the distribution of EMG data.

In addition to baseline period correction, within-subject z-standardization is often useful in fully within-subject EMG designs because it controls for stable individual differences in overall muscle reactivity [36]. However, in the present mixed design it creates a potential inferential problem. By centering each participant’s scores on that participant’s own mean, the transformation forces each participant’s average level to zero and thereby removes between-person differences in absolute activation. Because condition is a between-subjects factor in our paradigm, this step can attenuate the very condition-related variance needed to estimate condition main effects and interactions with distance. Faced with this trade-off, in Experiment 1 we chose to analyze square-root-transformed EMG amplitudes in the full condition × distance × muscle model. Artifacts such as coughing, yawing, sneezing, and so forth were cleaned manually. Trials with more than 1/3 missing data were removed completely. The overall percentage of missing EMG values was 1.3.

#### Results

Data analysis was performed in R/RStudio via the lmerTest extension [37] for lme4 [38] with robust standard error estimation via a sandwich estimator for fixed effect estimates (ClubSandwich, [39]. Note that this robustness does not apply to global testing (e.g., F test). All reported models are random-intercept-fixed-slope linear mixed models with condition [no hotspot vs hotspot] and distance (Location 0, 1, 2, 3) as fixed factors and id as cluster variable with a random intercept for id. Due to non-convergence, random slopes were not included. Global testing was done via F tests. As some of the factors included more than two levels, we used custom focused contrasts to test specific hypotheses (see [40].

##### Self-reported valence.

**Fig 3:**
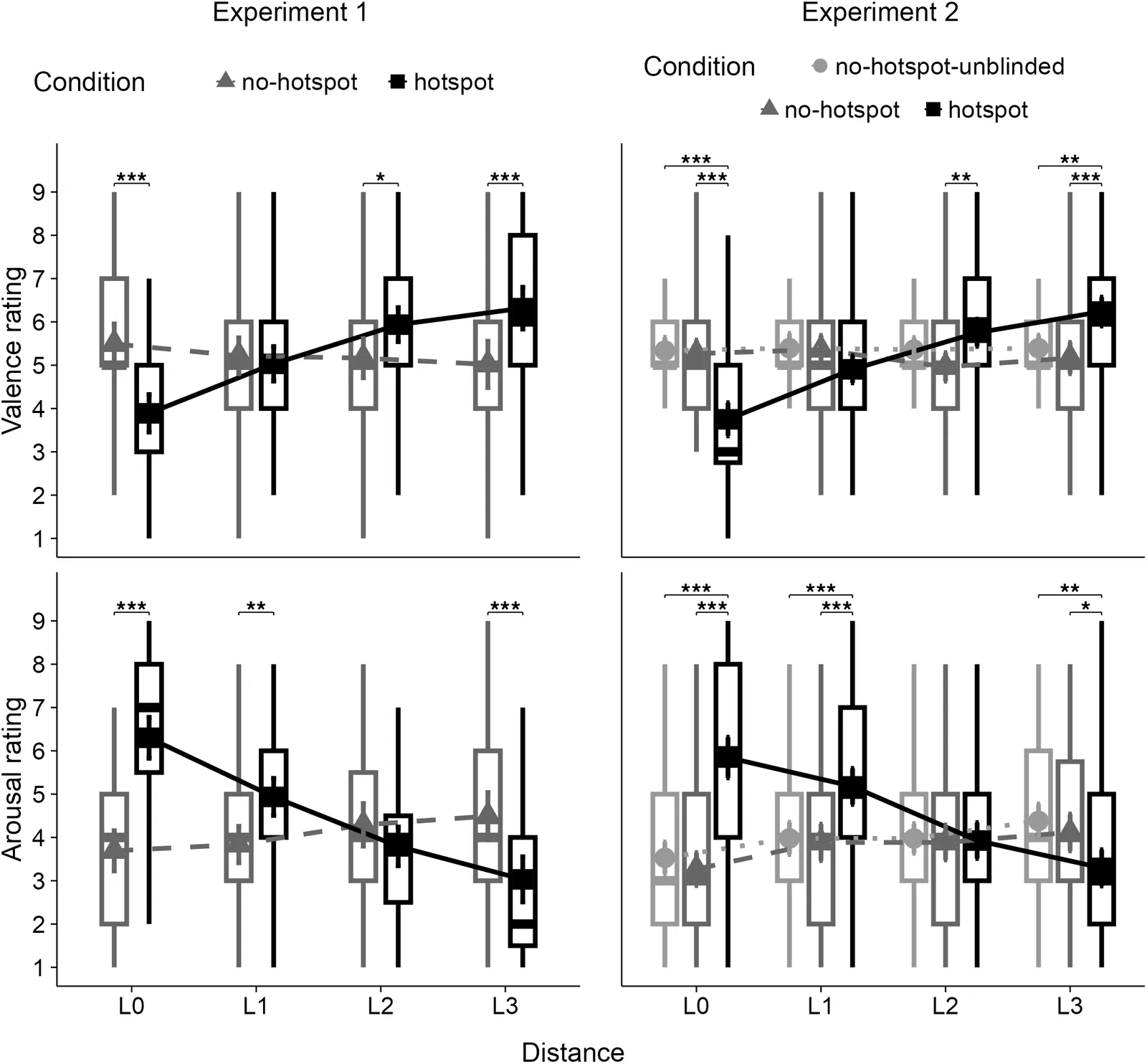
Self-reported valence (top) and arousal (bottom) as a function of condition and distance in Experiment 1 (left panel) and 2 (right panel). Note: Valence and arousal were rated via nine-point self-assessment manikins (SAM, [33]). Error bars represent 95% confidence intervals. Y-axis: Observed means. For estimated marginal means (emmeans, [41] of ratings), see R script report; X-axis: Distance with four locations (L0-L3).

An affective polarization of space effect would be indexed by the interaction between condition and distance so that self-reported positive feelings increase with increasing distance to the negative hotspot up to the point that a contrast effect emerges at the farthest distance. Indeed, there was a significant condition*distance interaction, *F*(1, 348) = 33.79, *p* < .0001, η_p_^2^ = 0.23. Focused contrasts indicated more negative valence ratings in the hotspot vs. no-hotspot condition at Location 0 (L0), *p*  <  .0001, Cohen’s *d*  =  1.35, 95% CI (0.82, 1.88), but not at Location 1 (L1), *p*  < .56, Cohen’s *d*  = 0.16, 95% CI [−0.37, 0.68]. A contrast effect (i.e., more positive valence ratings in the hotspot condition than in the no-hotspot condition) emerged already at location 2 (L2), *p*  < .031, Cohen’s *d*  =  −0.65, 95% CI [−1.18, −0.12], and intensified at L3, *p*  = .0002, Cohen’s *d*  = −1.09, 95% CI [−1.62, −0.56]. We additionally note that the often used pairwise comparisons (with less power) would indicate that the contrast effect emerged at L3 as predicted, *p*  = .0014, Cohen’s *d*  =  −1.09, 95% CI [−1.63, −0.56]. Overall, the affective polarization of space for self-reported valence that has been observed in previous studies generalized to the setting of Experiment 1. Fig 3 (left panel, top) shows the observed means (for estimated marginal means, see R script report).

##### Self-reported arousal.

The predicted condition*distance interaction was significant, *F*(1, 348) = 59.69, *p*  < .0001, η_p_^2^ = 0.34. All individual fixed effects estimates for the interaction were significant (*p*  <  .001, for details, see supplementary material). As expected, focused contrasts indicated higher arousal ratings in the hotspot vs. no hotspot condition at L0, *p*  <  .0001, Cohen’s *d* = −2.077, 95% CI [−2.62, −1.53], and L1, *p*  =  0.0025, Cohen’s *d*  = −0.875, 95% CI [−1.41, −0.34], as well as lower arousal ratings at L3, *p* = 0.0001, Cohen’s *d* = 1.161, 95% CI [0.63, 1.70]. Thus, affective polarization of space emerged in Experiment 1 for self-reported arousal as well. Fig 3 (left panel, bottom) shows the observed means (for estimated marginal means, see R script report).

##### Physiological activity in response to locations.

**Fig 4:**
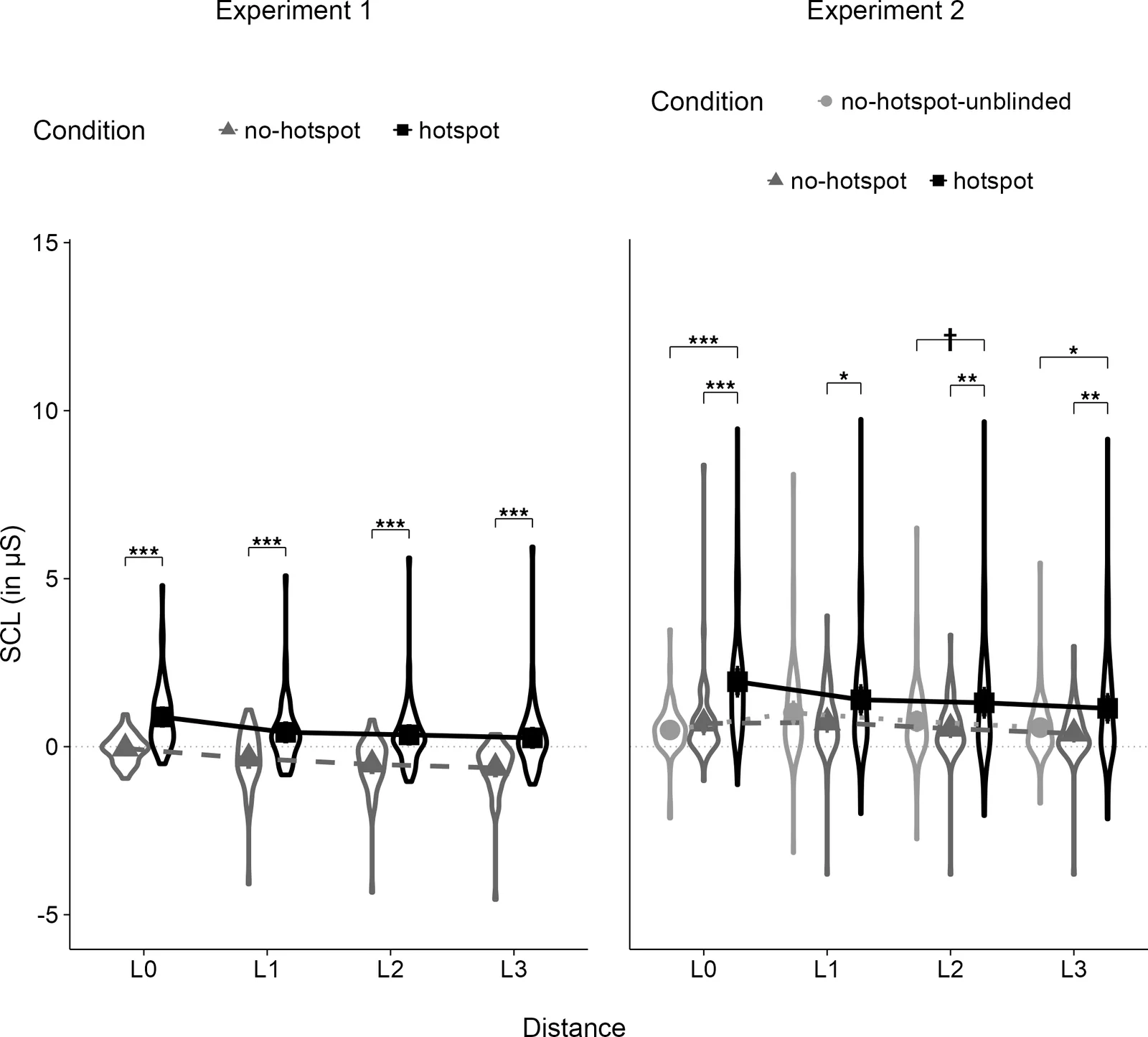
Skin conductance level as a function of condition and distance in Experiment 1 (left panel) and 2 (right panel). Note: Mean tonic skin conductance level was measured in microsiemens (µS) and was baseline-corrected (3-minute segment from static baseline subtracted).

***Electrodermal activity.*** There was no significant condition*distance interaction, *F*(1, 332) = 1.07, *p* = .363, η_p_^2^ = .009, but a significant main effect of distance, *F*(1, 112) = 89.16, *p*  <  .001, η_p_^2^ = .45, and a significant main effect of condition, *F*(1, 332) = 31.89, *p* < .001, η_p_^2^ = .22. As expected, SCL was higher in the hotspot condition than in the no-hotspot condition at L0, *p*  <  .0001, Cohen’s *d* = −2.95, 95% CI [−4.01, −1.90]. SCL remained higher in the hotspot condition at each distance, L1: *p*  <  .0001, Cohen’s *d* = −2.95, 95% CI [−3.99, – 1.92], L2: *p* < .0001, Cohen’s *d*  = −2.95, 95% CI [−3.99, −1.92], L3: *p*  < .0001, Cohen’s *d* = −2.95, 95% CI [−3.99, −1.92]. In the hotspot condition, SCL stayed above baseline level (marked by zero) throughout the experiment. In the no-hotspot condition, SCL was not different from baseline at L0 and lower than baseline at L1-L3. Thus, SCL indicates that the experimental manipulation was successful in inducing stress (i.e., more arousal at L0 in the hotspot condition), but it did not show the pattern of affective polarization of space found for self-reported arousal (i.e., less arousal at L3 in the hotspot condition). Fig 4 shows the observed means (for estimated marginal means, see R script report).

**Fig 5:**
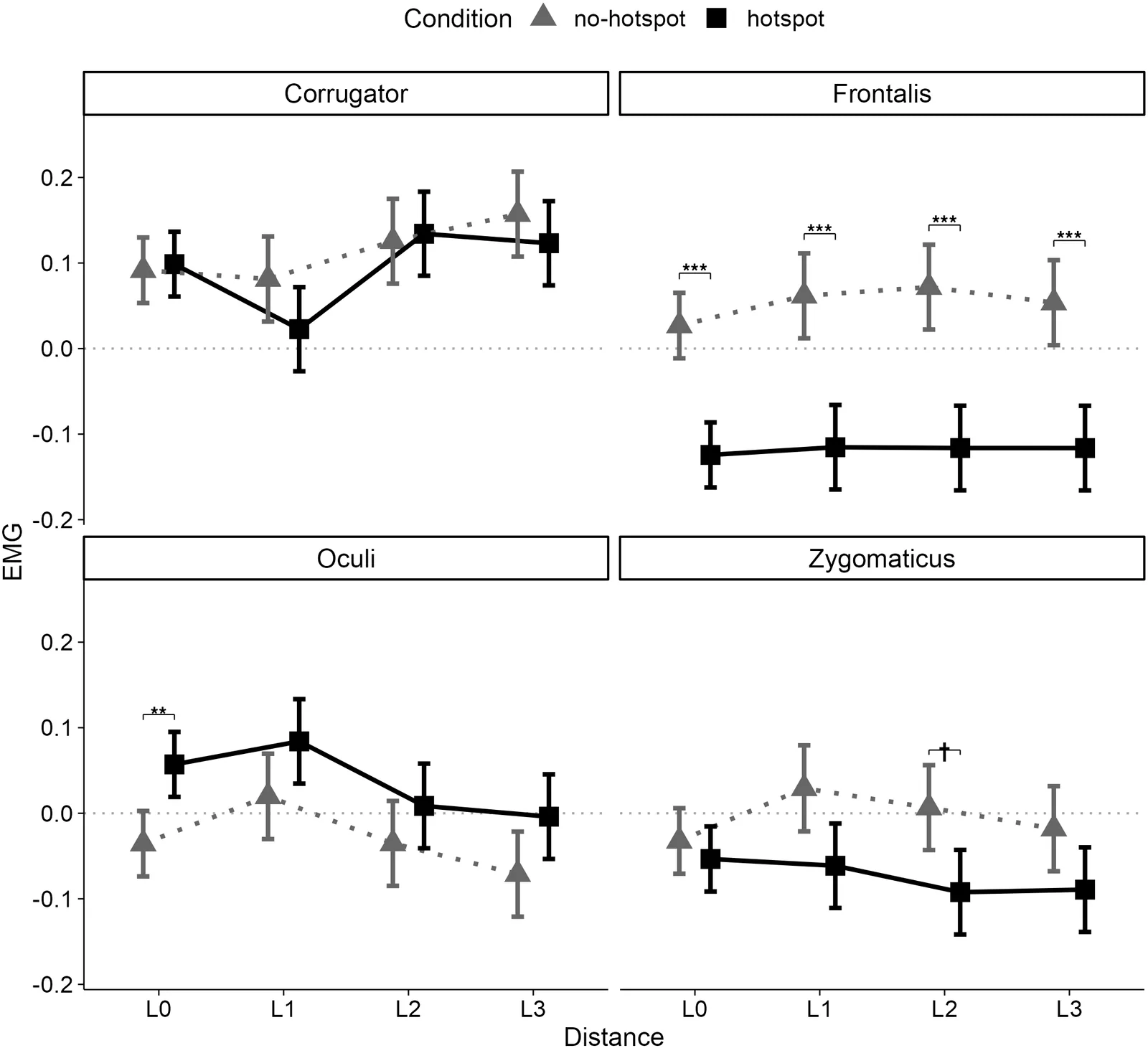
Facial activity as indexed by EMG channel scores as a function of condition and distance (response to spatial surroundings). Note: Corrugator = Corrugator supercilii, Frontalis = Frontalis pars medialis, Oculi = Orbicularis oculi lateralis, Zygomaticus = Zygomaticus major. L stands for location. Please recall that the EMG score (as the dependent variable) was baseline-corrected (with average across 90 seconds prior to end of static baseline) and square-root transformed (SQR) to counteract positive skew. Univariate outliers (e.g., implausible values) were treated with the adjusted outlyingness method [35]. Bars represent confidence intervals. Plot shows estimated marginal means; for observed means, see R script report.

***Facial electromyography.*** If facial activity differed according to condition and distance, we expect a significant three-way condition*distance*muscle site interaction. This interaction, however, was not significant, *F*(1, 2882) = 0.39, *p* = .93. Instead, there was only a significant condition*muscle site interaction, *F*(1, 2879) = 18.26, *p*  < .0001, η_p_^2^ = .02, with a very small effect size. The fixed effects estimates for both the full and the two-way interaction model indicated that the interaction was mainly driven by the frontalis muscle site (see Fig 5; for detailed results, see R script report). Frontalis activation was higher in the no-hotspot condition than in the hotspot condition, which is, however, more likely attributable to orienting behavior instead of a true indicator of apprehension or anxiety. At L0, orbicularis oculi was more active in the hotspot condition than in the no-hotspot condition, which is, however, more likely attributable to eye-crinkling due to apprehension than positive affect. Thus, the overall pattern of results was inconclusive and inconsistent with the pattern of affective polarization shown by the self-report measures.

### Discussion

The goal of Experiment 1 was to test whether the affective polarization of space robustly found with imaginary situations in previous vignette studies generalizes to a laboratory experiment involving an actual physical threat, and whether the effects extend to physiological responses indicative of the predicted affective responses. The results showed that the affective polarization of space found in previous work generalizes to the experimental context. Ecological validity was questionable in the previous work because vignettes were used rather than an actual experience. In the present research, participants’ skin conductance levels coupled with the subjective self- reports suggest that participants experienced some level of fear or apprehension near the negative hotspot. In this more ecologically valid situation, we replicated results obtained with vignettes: an affective polarization of space involving assimilation and contrast effects on the valence and arousal dimensions emerged. However, affective polarization of space was not mirrored in the physiological measures. Despite the powerful affect induction, neither facial EMG nor skin conductance reflected a complete pattern of affective polarization of space. Close to the hotspot, SCL indicated that the experimental manipulation was successful in inducing stress. However, neither skin conductance nor facial EMG revealed the unfolding of an affective polarization of space further away. Given the empirical support for facial EMG as an indicator of the valence and intensity of affective reactions (e.g., [19]), the absence of results in the present study is rather surprising.

### Experiment 2

#### Introduction

##### Affective polarization of space and affective empathy toward strangers.

Experiment 2 extended Experiment 1 by asking whether the affective structuring of space shapes responses to other people. If the affective polarization elicited by a hotspot changes how a surrounding location feels, it may also influence how people perceive and respond to strangers encountered there. Social interactions do not happen in a vacuum: the spatial context in which they occur may affect affiliation and empathy. Threat may cause fight-flight-freeze or tend-and-befriend responses, which influence social interactions (e.g., [42,43]. Threatening environments may trigger avoidance motivation, thereby reducing the tendency to affiliate and empathize with others. Conversely, greater distance from the threat may elicit relief and restore affiliative motivation. Alternatively, threat may increase the desire to affiliate, particularly with others who may share the same threat [44]. Findings such as men being more likely to be empathic and prosocial after a stressful episode [45] are consistent with this possibility. In Experiment 2, we therefore examined two indicators of affective empathy toward strangers: social warmth measured by self-report [46,47] and emotional mimicry as indexed by rapid facial responses to emotionally expressive faces [48,49].

##### Social warmth.

Social warmth is a fundamental dimension of social cognition and a natural indicator of affective empathy [50]. Alongside competence, warmth constitutes one of the two universal axes along which people evaluate others [46,47]: it reflects perceived benevolence and care, tends to dominate early social impressions, and is weighted heavily in approach and affiliation decisions. As an indicator of affective empathy, warmth captures the degree to which a perceiver feels positively engaged and affiliatively inclined toward a target [50]. Because social warmth is sensitive to the motivational context in which social encounters occur, it should be modulated by incidental affect [51]. Close proximity to a threatening hotspot is expected to activate avoidance motivation, which would reduce warmth toward strangers encountered there; conversely, the relief associated with greater distance from the hotspot should reinstate affiliative motivation and increase warmth. People can report how warm they feel toward a target person with a “feeling thermometer” [52], drawing on the conceptual link between physical temperature and social warmth [53,54].

##### Emotional mimicry.

Emotional mimicry is also an indicator of affective empathy [55–57]. Emotional mimicry (for reviews, see [48,49] refers to the spontaneous, rapid, automatic, but goal-dependent response to an interaction partner’s emotional display (e.g., [55,56]. Emotional mimicry is typically assessed as facial mimicry, the facial response to emotional displays. Emotional mimicry is related to subjective reports of affective empathy [57–59], and predicts the perceived quality of social interactions [60–62]. According to the mimicry as social regulator view [48,49], emotional mimicry is driven by as well as conducive to affiliation. Emotional mimicry is sensitive to contextual top-down modulations (e.g., [63–65]; for a review, see [49]. One class of context effects that are directly relevant is the affective context [66–70]. For instance, when emoters are embedded into affective scenes, participants mimic emoters whose facial expression is incongruent with the affective tone of the scene (e.g., a large smile at a funeral) to a lesser extent [66]. Showing an expression in an affectively incongruent context signals emotional deviance [71], which in turn disrupts the affiliation motive and thus emotional mimicry. Hence, if a threatening context reduces empathy, one would expect reduced mimicry as well.

##### Hypotheses.

In Experiment 2, participants were exposed to the same environment as in Experiment 1 but also saw actors expressing different facial emotion expressions at each location. We hypothesized that, compared to the control condition, an affective assimilation effect would emerge such that participants report significantly lower social warmth toward the actors when they are close to the hotspot. We hypothesized further that, compared to the control condition, a contrast effect would emerge such that they report significantly higher social warmth toward the actors farther away. Concerning emotional mimicry, the predictions were more exploratory as different processes could be in play. In line with the predictions for social warmth and with emotional mimicry in social context theory [56], stress caused by the threatening hotspot close by could disrupt affiliation and decrease emotional mimicry, particularly for the affiliative emotions (happiness and sadness, whereas this would not be the case for anger, which is likely to be perceived as antagonistic in this context). In contrast, relief due to greater distance from the hotspot could leave emotional mimicry, again particularly for the affiliative emotion, undisturbed or even enhance it (comparison to control condition; contrast effect). In line with a valence account, given the notion of congruence in affective tone, a likely outcome could also be that, compared to the control condition, happiness mimicry would be reduced close to the threatening hotspot and stronger further away, and the other way round for the mimicry of negatively valenced emotions (sadness and anger). Finally, a less likely but possible alternative could be that the immediate threat close to the hotspot could also enhance affiliation and hence mimicry due to the potential increase of re-affiliation under conditions of stress [44] or the potential for appeasement when faced with a threat.

#### Method

##### Participants.

A total of 254 different healthy volunteers (130 women and 124 men) in the age range between 18 and 45 years (*M* = 28.9, *SD* = 6.83) participated in Experiment 2. Of these, 23.2% were psychology students, 39.4% were students from other fields and 36.2% were non-students, whereas 1.2% did not report this information. Ethical approval was provided by the local institutional review board (#2015−30) of the Institute of Psychology of the Humboldt-Universität zu Berlin and written informed consent was obtained from all participants. The data collection went from 8/11/2017–8/10/2019.

##### Procedure.

The procedure of Experiment 2 was the same as in Experiment 1, except for the following differences. The mean shock-tolerance threshold in this sample was 46.88 V (*SD* = 16.14). Mean temperature was 22.9 (*SD* = 1.47) and mean humidity was 41.26 (*SD* = 10.1). In addition, we recorded cardiac activity (from ECG) via a lead-II configuration (the ground electrode was placed at the left clavicle, the negative lead was placed at the right clavicle, and the positive lead was placed below the left rib cage). Due to the ECG recording, a longer, i.e., six-minute version of the baseline video of Experiment 1 was used. Initial analyses of Experiment 2 pointed toward a confound in the control group. Keeping participants in the no-hotspot group blind until debriefing at the very end of the session may have left them with more apprehension than intended. When participants in the no-hotspot condition do not know what will occur except that they may receive shocks—which had to be disclosed for basic ethical reasons—they are left wondering whether they will in fact be shocked. Participants in this condition were often surprised when the experiment ended without them receiving any shocks. When participants expect to be in the experimental condition (as in expectancy-based placebo effects; [72], this uncertainty may leave them with residual apprehension or anticipatory anxiety. This could also explain the lack of assimilation and contrast effects for the physiological measures in Experiment 1. Therefore, we introduced a third group post hoc. In that second control group, participants were unblinded right away before starting the main experiment to mitigate any residual apprehension or anticipatory anxiety. As this post hoc control group was introduced later in time (i.e., the no-hotspot-unblinded condition was collected from 07/31/2019–10/08/2019, whereas the other two conditions were collected before 05/19/2018), it cannot be excluded that time of data collection influenced the results. However, the main and interaction effects involving the main factors condition, distance, and muscle remained present after controlling for elapsed days. For the sake of simplicity, we therefore kept the simpler models (see the ancillary analyses in the R script reports). In the following, we will label the condition with the negative hotspot the hotspot condition, the no hotspot group with delayed unblinding the no-hotspot condition, and the no hotspot group with the immediate unblinding the no-hotspot-unblinded condition (see Fig 1).

In the test phase, the participants were instructed that they will encounter passersby at each of the three remote locations. There, they saw four male faces from the FACES database [32], embedded into the scenes (original blank background removed, then superimposed). Each passerby’s face displayed one of four different emotional facial expressions (happy, sad, angry, and neutral). The passersby and their facial expressions appeared in a different random order for each participant. Within a remote location, each of the four faces appeared for 5 s with an inter-trial interval of 2 s. In the rating phase, participants were instructed that they would see the passersby again at the location where they encountered them before. Each face was followed by a feeling thermometer rating for the assessment of social warmth toward the passersby. The whole sequence was followed by self-reports on relative attentional focus on scene vs. face (participants reported to be focused more on face overall, *M* = 72.6% on 100% slider scale from scene to face; but they were more focused on place in hotspot group at location 1, *M*  = 66.1, than in the no-hotspot-unblinded group, *M* = 78.6, *p* = .014), valence (*M* = 2.96, *SD* = 1.24, 9-point scale) and arousal (*M* = 7.55, *SD* = 1.13, 9-point scale) of the shocks, and estimated likelihood of receiving a shock (i.e., getting “assaulted”) at each remote location (location 1: 58.6, location 2: 38.9, location 3: 23.3), and their cognitive load due to the shocks (on 0–100 slider scale location 1: 66.4 location 2: 55.2, location 3: 37.0). Whereas these indices all suggested the meaningfulness of the manipulation, we still considered it crucial to add this additional control group to the experiment.

##### Measures.

Experiment 2 entailed the same subjective ratings of valence and arousal, as well as the same measure of electrodermal activity as in Experiment 1. We describe here the measures that are specific and central to Experiment 2.

###### Open response.

The participants were asked to write a few sentences to explain in their own words why they rated Location 3 on the valence and arousal scales as they did. This measure potentially taps into the phenomenological experience of the participants at Location 3. To analyze the emotional content of these responses as a function of condition, we parsed the participants’ generated content with Klaus Scherer’s Geneva Affect Label Coder (GALC; [73]. GALC is a lexicon- and stem-based coding tool for free reports of affective experience based on Excel macro: it searches and counts written responses for affect-related words, word stems, and synonyms in English, French, and German, and maps these matches onto 36 discrete affect categories (e.g., delighted, desperation, confident, or relaxed). Following Scherer’s coding logic, occurrence of a term belonging to a category family is treated as evidence for presence of that affect category in the response. The method is sensitive to explicit emotion vocabulary, but does not fully resolve negation, intensity, irony, or figurative language. We used GALC rather than a generic sentiment-analysis approach because our aim was not to estimate overall positive or negative sentiment, which was already assessed through valence ratings, but to identify which specific affective states participants spontaneously invoked when explaining their ratings of Location 3.

###### Social warmth.

The participants rated how much warmth they felt toward the emoter by using a feeling thermometer that ranged from 0 = cold, rejecting feelings to 100 = warm, benevolent feelings.

###### Electrodermal activity.

The same EDA data preparation as in Experiment 1 was used in Experiment 2. The last 3 m of data were taken from the 6 m baseline procedure.

###### Cardiac activity.

Electrocardiography (ECG) was continuously recorded at a sampling rate of 1,000 Hz. The skin was cleansed with rubbing alcohol and two prejelled Mindware Ag/AgCl snap disposable vinyl electrodes were placed on the participants’ right collarbone and left lower rib and one prejelled Mindware Ag/AgCl snap disposable vinyl reference electrode was placed on participants’ right lower rib. A bandpass filter of 0.5 Hz–100 Hz (and a 50 Hz notch filter) was used, and the ECG signal was converted into R-wave intervals. Artifacts and recording errors were corrected manually.

###### Emotional mimicry.

The same EMG data preparation as in Experiment 1 was used in Experiment 2 with one difference. The EMG data were this time not simply square-root transformed but within-subject z-standardized because we wanted to measure patterns of muscle activation that are associated with specific facial expressions (emotional mimicry), not just positive or negative affect. In this case, it is even more important to control for stable differences in muscle reactivity with a within-z standardization, as it is usually recommended to combine these muscle activation into pattern scores (i.e., contrast indices) that reflect particular facial expressions [19]. Yet, due to our mixed-design, we were confronted with the same condition effect attenuation problem as in Experiment 1. Faced with this trade-off, we chose to perform the within-z transformation for two reasons. First, we judged that the variance caused by computing contrast indices without within-subject z-standardization per muscle would be more detrimental than the potential attenuation effect due to the within-z standardization. Second, we opted for a simpler condition x distance analysis design for each emotion separately, which attenuates the interpretation problems caused by within-subject z-standardizing muscles across different emotions that reflect different underlying mechanisms. The overall percentage of missing EMG values was 3.4.

###### Eye tracking.

Using a Tobii TL-60, we measured time spent in different areas of interest, namely face, place (the location, i.e., the rest of the image minus the face and map), and map. This measure was solely used for auxiliary analyses.

#### Results

We used random-intercept-fixed-slope linear mixed models with condition (no hotspot, no hotspot-unblinded, hotspot) and distance (L0, L1, L2, L3) as fixed factors and id as cluster variable with a random intercept for id. The dependent variables were self-reported valence and arousal, respectively. For full analysis details, see R script report.

##### Self-reported valence.

There was a significant condition*distance interaction, *F*(1, 744) = 29.55, *p*  < .0001, η_p_^2^ = 0.19. At L0, focused contrasts showed more negative valence ratings in the hotspot vs. no hotspot condition, p < .0001, Cohen’s *d*  = 1.37, 95% CI [0.95, 1.80], and vs. the no hotspot-unblinded condition, *p* < .0001, Cohen’s *d*  = 1.44, 95% CI(1.02, 1.85), but not at L1, *p*  < .33, Cohen’s *d* = 0.41, 95% CI [−0.01, 0.83], and *p*  <  .23, Cohen’s *d* = 0.44, 95% CI [0.04, 0.85], respectively. More importantly, the expected contrast effect was significant, with the hotspot group reporting more positive valence at L3, than the no-hotspot group, *p* = 0.0001, Cohen’s *d* = −0.97, 95% CI [−1.39, −0.55], and the no-hotspot-unblinded group, *p* = 0.0022, Cohen’s *d* = −0.76, 95% CI [−1.17, −0.36]. The contrast effect emerged already at L2, for hotspot vs. no hotspot, *p* = .0072, Cohen’s *d* = −0.71, 95% CI [−1.13, −0.29]. However, no difference in self-reported valence between the no hotspot and the no hotspot-unblinded condition emerged at any location (all *p* > .44). Overall in Experiment 2, similar as in Experiment 1, there was affective polarization of space in self-reported valence as can be seen in Fig 3 (right panel, top).

##### Self-reported arousal.

There was a significant condition*distance interaction, *F*(1, 744) = 45.89, *p* < .0001, η_p_^2^ = 0.27. At L0, focused contrasts indicated higher arousal ratings in the hotspot condition vs. the no hotspot condition, *p* < .0001, Cohen’s *d* = −2.23, 95% CI [−2.73, −1.72], and vs. the no hotspot-unblinded condition, *p* < .0001, Cohen’s *d* = −2.00, 95% CI [−2.49, – 1.52]. At L1, there were higher arousal ratings in the hotspot condition vs. no hotspot condition, *p* = 0.0001, Cohen’s *d* = −1.12, 95% CI [−1.62, −0.62], and vs. the no hotspot- unblinded condition, *p* = 0.0002, Cohen’s *d* = −1.04, 95% CI [−1.52, −0.56]. In contrast, arousal ratings at L3 were lower in the hotspot condition vs. the no hotspot condition, *p* = 0.037, Cohen’s *d* = 0.71, 95% CI [0.21, 1.20], and vs. the no hotspot-unblinded condition, *p* = 0.0011, Cohen’s *d* = 0.94, 95% CI [0.45, 1.42]. No differences in self-reported arousal emerged between the no hotspot and the no-hotspot- unblinded condition at any location (all *p* = 1.0000). Thus, as in Experiment 1, the results of Experiment 2 indicated that the affective polarization of space for self-reported arousal emerged as can be seen in Fig 3 (right panel, bottom).

##### Open response.

For each participant’s open responses, the GALC Excel macro coded whether each observed GALC category was present and we then compared category prevalence across conditions (no hotspot, no hotspot-unblinded, hotspot; *n* = 89, 78, and 82). In total, 15 GALC categories were observed in the sample. For each observed category, we tested a 3 × 2 contingency table (condition × category present/absent). Because many expected cell counts were below 5, which is a condition under which the chi-square approximation is unreliable, we used Fisher’s exact test extended to r × c tables via Monte Carlo simulation (*B* = 10,000; with simulate.p.value set to TRUE in R’s fisher.test()), as recommended for computationally intensive exact tests [74]. *p*-values were adjusted for multiple comparisons using the Benjamini-Hochberg (BH) false discovery rate procedure.

After correction, only one GALC category differed significantly between conditions: the word “relaxation” (“Entspanntheit/Entspannung” in German; hotspot = 18 participants, no hotspot = 3, no hotspot-unblinded = 2), Fisher’s *p* < .001, *p*_BH_ < .001. All other categories, including “positive” (Fisher’s *p* = .021, *p*_BH_ = .16), did not reach significance after correction. The fact that many more participants in the hotspot condition spontaneously evoked feelings related to the GALC category “relaxation” at Location 3 supports the notion that a genuine affective polarization of space emerged at the phenomenological level.

##### Physiological activity in response to locations.

###### Electrodermal activity.

There was a significant condition*distance interaction, *F*(1, 660) = 20.05, *p* < .0001, η_p_^2^ = 0.15. In Experiment 2, all SCL values were higher than baseline, unlike in the no-hotspot condition in Experiment 1. Overall, as in Experiment 1, the pattern of sympathetic arousal in Experiment 2 is compatible with a successful manipulation. SCL in the hotspot condition was higher at L0 than in the other two conditions, for no-hotspot: *p* < .0001, Cohen’s *d* = −2.87, 95% CI [−3.87, −1.87], and no-hotspot-unblinded: *p* < .0001, Cohen’s *d* = −3.15, 95% CI [−4.12, −2.18]. Thus, participants in the hotspot condition appeared to experience more sympathetic arousal in the induction phase (i.e., the hotspot) than participants in the other two conditions. As in Experiment 1, there was no indication of an affective polarization of space at the autonomic activation level. In the present experimental context, however, changes in SCL are most plausibly interpreted as consistent with heightened bodily mobilization (e.g., increase in sweat gland activity that can support action readiness) to the threatening and spatially immediate aspects of the situation, which weakened as the threat dissipated.

###### Cardiac activity.

**Fig 6:**
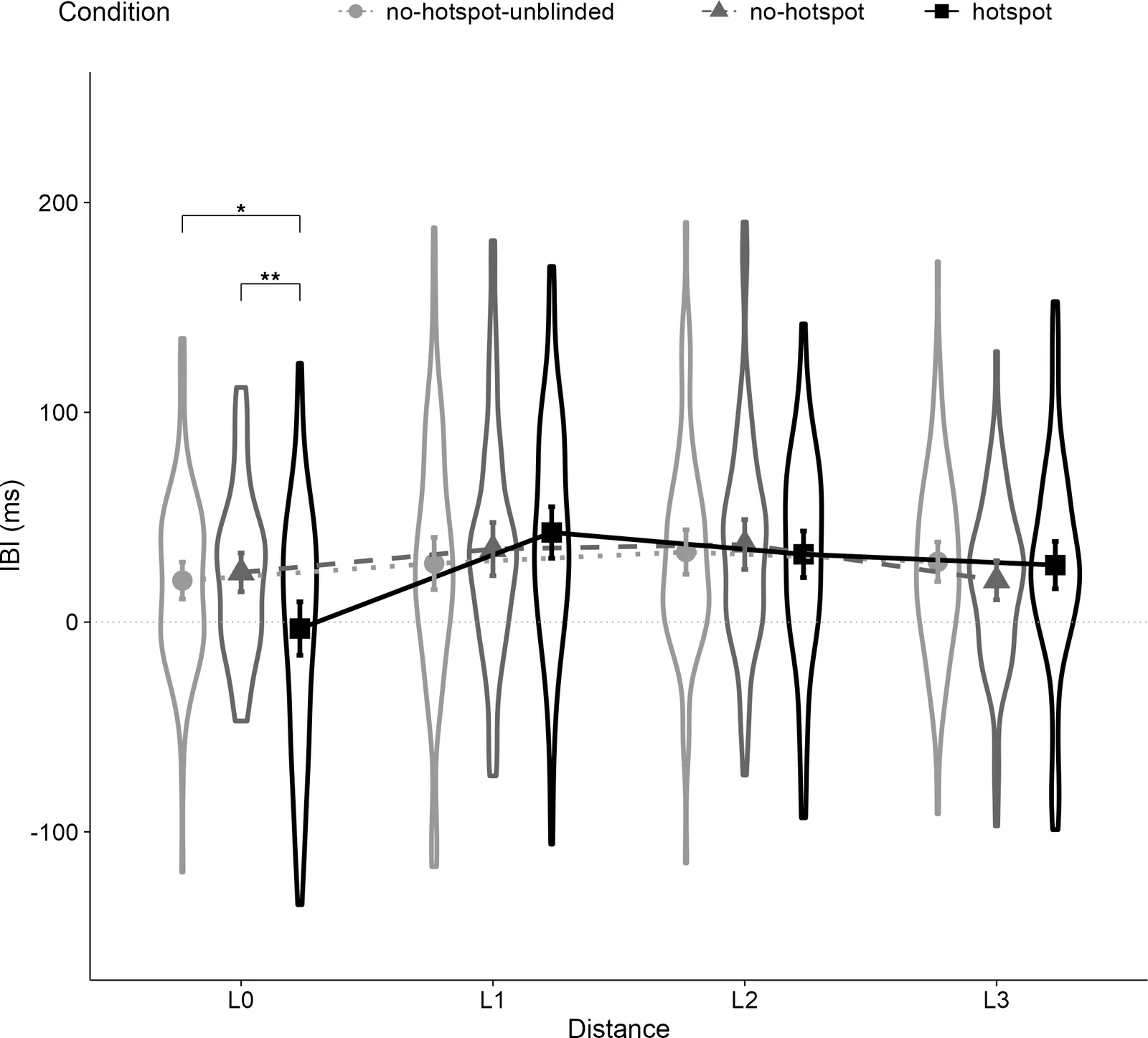
Cardiac activity as indexed by mean interbeat interval (IBI) as a function of condition and distance in Experiment 2. Note: Mean interbeat interval is measured in milliseconds (ms) and was baseline-corrected (difference score with a 90-second segment prior to end of static baseline subtracted).

There was a significant condition*distance interaction, *F*(1, 694) = 7.87, *p* < .0001, η_p_^2^ = 0.07. However, the difference in mean interbeat interval (IBI)—although in the expected direction with IBI lower in the hotspot condition than in the no hotspot condition—failed the conventional significance level, *p* = .069, Cohen’s *d* = 0.8098, 95% CI [0.3204, 1.2991]. Nevertheless, the difference in IBI between the hotspot condition and the no hotspot-unblinded condition was significant, *p* = 0.038, Cohen’s *d* = 0.9012, 95% CI [0.39, 1.42]. Focused contrasts with fewer comparisons, revealed both comparisons to be significant (*p* = .013, *p* = .008, respectively). As such, the data supports the conclusion that a higher heart rate, emerged in the hotspot condition at L0. Thus, the heightened cardiac activity closer to the threat, together with skin conductance and the self-report measures of affect, provides converging evidence for a successful threat manipulation. However, as for skin conductance, there was no indication of affective polarization of space. Moreover, it has to be noted that in the hotspot condition, heart rate as indexed by IBI was not higher than baseline at L0. For the observed means, see Fig 6 (for estimated marginal means plot, see R script report).

##### Affective empathy.

To assess whether affective judgment in spatial context had an effect on indicators of affective empathy, we measured self-report of cold or warm feelings and EMG-based emotional mimicry toward passersby in the surroundings of the negative hotspot. We used random-intercept-fixed-slope linear mixed models with condition [hotspot vs no hotspot vs no hotspot-unblinded], distance (Location 0, 1, 2, 3), and emotional facial expression (happy vs sad vs angry) as fixed factors and id as cluster variable with a random intercept for id in the case of social warmth. We analyzed each emotion separately in the case of emotional mimicry. For full analysis details, see R script report.

###### Self-reported social warmth.

Reported here are the results for the emotional faces corrected with a neutral expression (results led to the same conclusion as in the uncorrected case). For self-reported social warmth (feeling thermometer ratings), there was only a significant main effect of emotion, *F*(1, 602) = 601.59, p < .0001, η_p_^2^ = 0.37. Based on pairwise comparisons, participants had warmer feelings toward happy facial expressions than toward sad expressions, *p* < .0001, Cohen’s *d* = 0.807, 95% CI [0.70, 0.91], which, in turn, elicited more warmth than angry expressions, *p* < .0001, Cohen’s *d* = 0.98, 95% CI [0.88, 1.08]. Thus, the effect of facial expression was greater than any effect of condition or distance, which were both non-significant. This finding was supported by the auxiliary eye tracking data. These indicated that this effect was independent of a difference score of face minus place minus map. Moreover, participants looked in the face most of the time, *F*(1, 950) = 3106, *p* < .0001, η_p_^2^ = 0.76, which reflects the style of analytic cognition that can be expected from such a sample [75].

###### Emotional mimicry.

To count as an emotional mimicry response, the estimated marginal means had to be different from zero, for the respective emotion, condition, and location. Again, analyses were conducted separately for each emotion as the expected expressive pattern differs between emotions and, for example, sadness and happiness mimicry may underlie different processes [48,52]. Mimicry was assessed based on Corrugator S., O. Oculi, and Zygomaticus M. Happiness mimicry is evidenced by increased O. Oculi and Zygomaticus M. and decreased Corrugator S. activity, sadness and anger mimicry by the converse pattern. One way to capture these predicted patterns of expression in a single metric is to calculate a contrast score in which Corrugator S. is subtracted from the mean of O. Oculi and Zygomaticus M. [19]. A negative score indexes a sad expression, whereas a positive score indexes a happy expression.

**Fig 7:**
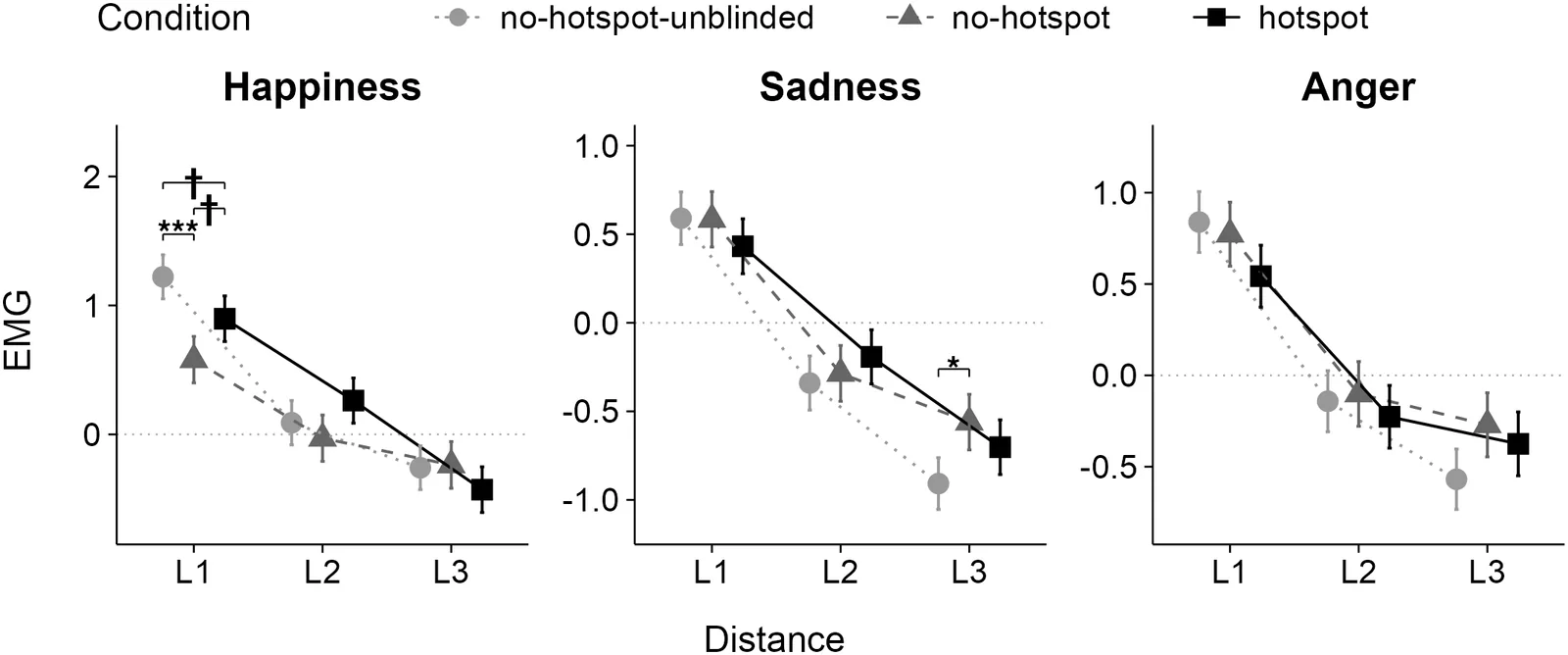
EMG scores in response to happy, sad and angry expressions as a function of condition and distance (in separate analyses). Note: The EMG score was baseline-corrected (90-seconds prior to static baseline segment) and within-subject z-standardized. Positive scores indicate facial activity that is higher than baseline and higher zygomaticus and oculi than corrugator. Outliers were treated with the adjusted outlyingness method [35]. Bars represent 95% confidence intervals. Plot shows estimated marginal means; for observed means, see R script report.

For the response toward happy faces, there was a small but significant condition*distance interaction, *F*(1, 3250) = 9.75, *p* < .0001, η_p_^2^ = 0.01. At L1, focused contrasts indicated a larger pattern score in the no-hotspot-unblinded condition than in the no-hotspot condition, *p* < .0001, Cohen’s *d* = 0.49, 95% CI [0.30, 0.67], whereas the difference between the no-hotspot-unblinded condition and the hotspot condition failed to reach the conventional significance level, *p* = 0.076. As can be seen in Fig 7 (left panel), estimated marginal means for all conditions suggested that whereas happy faces were mimicked at L1, happy faces were counter-mimicked at L3 (for observed means plot, see R script report). Happy faces were mimicked at L2 in the hotspot condition.

For sadness, there was a significant condition*distance interaction, *F*(1, 3236) = 3.72, *p* = .005, η_p_^2^ = 0.005, again with a small effect size. There appeared to be counter-mimicry at L1, and sadness mimicry at L2 and L3 in all three conditions. Focused contrasts showed that only no-hotspot-unblinded and the no-hotspot condition yielded statistically significantly different scores, *p* < 0.014, Cohen’s *d* = −0.27, 95% CI [- 0.4428, −0.10448]. For estimated marginal means plot, see Fig 7 (middle panel; for observed means plot, see R script report).

For anger, there was a significant condition*distance interaction, *F*(1, 3266) = 3.14, *p* = .014, η_p_^2^ = 0.004, again with a small effect size. Focused contrasts showed that there was no significant difference across conditions. There appeared to be again counter- mimicry at L0 and anger mimicry (or congruent negative reactions to the face) at L3. Counter-mimicry evidence at L2 was found only for the no-hotspot unblinded and the hotspot condition, for the no-hotspot condition the estimated marginal mean fell short of being significantly different from zero. For estimated marginal means plot, see Fig 7 (right panel; for observed means plot, see R script report).

Overall, it appears that participants showed a positive reaction to faces at the first location after the induction phase relatively independent from expression. With increasing distance, this facial positivity dissipated or even turned into negativity. This suggests novelty effects that turned into habituation or drift effects. Therefore, it is unclear whether actual reactions to the faces occurred. Moderation analyses using traits from the questionnaire battery as continuous moderators (trait empathy, anxiety, approach-/avoidance motivation, holistic mindset), heart rate variability (RSA, RMSSD), or self-reported social warmth, yielded no significant results and are not further reported here.

#### Discussion

Experiment 2 replicated the affective polarization of space for self-reported valence and arousal found in Experiment 1. The open response data supported the notion that a genuine affective polarization of space emerged at the phenomenological level, with participants in the hotspot condition spontaneously evoking “relaxation” at Location 3 more often. As in Experiment 1, the physiological measures did not show a complete pattern of affective polarization of space. Skin conductance and cardiac activity indicated a successful threat manipulation at L0, but neither revealed the unfolding of affective polarization further away. Concerning affective empathy, we hypothesized that the affective tone elicited by the hotspot in the surroundings (i.e., the affective field) would influence the amount of empathy felt toward passersby. For self-reported social warmth, the effect of facial emotion was much greater than the effect of the affective tone of the background. Future research may thus study the biasing effect of hotspots on perceived social warmth with more ambiguous social stimuli where ceiling effects are less likely. Paralleling the results for perceived social warmth, there was no clear difference in the extent of emotional mimicry between conditions (p-value below  .05 for the comparison between the hotspot and the no-hotspot condition but p-value only below  .10 for the comparison between the hotspot and the no-hotspot-unblinded condition). Yet, emotional mimicry of happy expressions at the first location was obtained across conditions. This speaks for the robustness of happiness mimicry, even under boundary conditions. Smiles in the safest experimental condition (no hotspot unblinded) elicited slightly more mimicry than in the latent threat condition (no hotspot); the difference with the manifest threat (hotspot) condition failed to reach significance. Similar patterns were obtained for sadness and anger at the first location. Yet, the similarity of participants’ facial activity across different expressions makes it less likely that the EMG responses represent mimicry. Emotional mimicry is more likely when the goal is to communicate understanding in order to affiliate with others [60]. Here, however, participants passively viewed unknown passersby without any explicit prompt to affiliate or to process their facial expression. Thus, only smiles, which are potent affiliation signals that are mimicked with minimal affiliative intent [76,77], appeared to elicit some potential differences in mimicry across conditions (and only at one location). Here, future research could also specifically take into account that people may engage in approach behavior toward close others but show more avoidant or indifferent behaviors toward strangers under conditions of threat. The threat of being shocked probably occupied participants’ attention, which further distracted participants from affiliating. Future studies about the influence of the affective field on empathy should strengthen participants’ affiliation motive such that it focuses more attention.

## General discussion

The two experiments reported here generalize and extend the results from previous work about affective judgment in spatial context (for a review, see [16]. In both experiments, affective polarization of space emerged for self-reported valence and arousal when participants experienced an actual physical threat in a laboratory setting. These results show that affective polarization of space is not only compatible with general principles of judgment in social cognition (e.g., [17]; see [16], but also with theories about the emergence of affect. According to Carver and Scheier’s cybernetic model of self-regulation [78,79], affect checks how well people do in approaching desirable goals or in avoiding undesirable ones. Similarly in our case, being close compared to being far away from the threatening hotspot is like faring poorly toward the goal of avoiding an undesirable goal. Therefore, participants reported experiencing negative affect with high arousal, which corresponds to emotions like fear or anxiety [80]. Reversely, being far away compared to being close to the threatening hotspot is like faring well with the avoidance goal. Therefore, participants reported experiencing positive affect with low arousal, like serenity, or relief. Our results thus support Carver and Scheier’s view that when a situation engages the avoidance behavioral system, the related affective dimension ranges from anxiety to relief [78]. Carver and Scheier’s framework predicts that affective polarization should extend to scenarios where individuals gradually distance themselves from a real threat, like here. However, it is uncertain if this effect also generalizes to opposite circumstances, such as when individuals gradually approach a real threat. Thus, a more careful interpretation of the results is that while they bolster the external validity of previous research about the affective polarization of space effect, they may only pertain to the case where participants gradually increase distance from a real threat. One implication of the results is that participants did not base their self-report of affect on their absolute level of physiological activation. This divergence may be due to the different time scales of the subjective affective judgment and the physiological processes. The mental realization that one is relatively safe compared to a previous condition may be faster than physiological recovery, as when one wakes up from a nightmare with relief while still being highly aroused physiologically. Relief thus comes first and is followed by physiological recovery. In this perspective, our results are explainable. In general, it is important to note that physiology and subjective experience can often be related but by no means move in lockstep as associations of physiology with experience and behavior are often only modest [81–84].

Several limitations apply to our study. Due to practical reasons of measurement, we used three locations in space at increasing distances (four discrete levels). A cautious interpretation should restrict the conclusions to the specific operationalization employed in this study. A different limitation is that participants did not report their affect during the stimulus presentation but shortly after. Retrospective report of affect is based on episodic memory, not current feelings [85]. However, a few seconds only separated the actual affective experience from the report so that contextual details were still available to reconstruct a reliable emotional experience [85]. A further limitation concerns the ecological representativeness of the test stimuli. For better control of systematic and unsystematic error variance caused by any salient, colored, objects in the environment, the remote locations were depicted as empty gray-scaled environments with only the towers of the hotspot visible in the distance. Thus, they do not capture the complexity of real urban environments, which limits the generalization of the present findings to richer spatial contexts. Despite these limitations, the results presented here are closer to actual affective experiences in real life than previous results based on vignettes. The fact that both methodologies produce similar results suggests that affective polarization of space is more than a prospective bias but rather a genuine phenomenon rooted in people’s online affective experience within physical space. The threat induction itself, combining first-person perspective, threatening video footage, and actual electrical stimulation, is also a design strength in its own right: rather than asking participants to project themselves into an imagined scenario, it engaged their affective system in real time, and the elevated skin conductance near the hotspot supports the interpretation that participants were indeed genuinely afraid. Future research could further test the external validity of these findings by placing participants in immersive virtual reality environments or actual physical settings, which would allow manipulation of distance in a way that is even closer to real-world threat experiences.

## Conclusion

In two laboratory experiments, we gathered evidence that the affective polarization of space is rooted in emotional experience in the here and now. Specifically, we were able to replicate the pattern of assimilation and contrast in self-reported valence and arousal found in previous vignette studies conducted online. When closer to a threatening hotspot, participants usually report an affective state comparable to that of the hotspot (negative valence, high arousal), whereas farther away they report an affective state that is indicative of contrast or relief. However, this self-reported affective polarization was not mirrored by the physiological measures and had no consequences for the extent of displayed affective empathy toward passersby. Thus, whereas the present evidence advances the theory of affective judgment in spatial context [13], it also suggests possible methodological avenues for studying the effect of the affective field on social cognition and on physiological manifestations.
